# Risk factors of bicycle traffic injury among middle school students in chaoshan rural areas of china

**DOI:** 10.1186/s12939-016-0512-8

**Published:** 2017-01-26

**Authors:** Zhen-bin Lin, Yan-hu Ji, Qing-yu Xiao, Li-bo Luo, Li-ping Li, Bernard Choi

**Affiliations:** 1 Shantou University Medical College, Shantou, China; 2 Joint Shantou International Eye Center of Shantou University and the Chinese University of Hong Kong, Shantou, China; 3 Dalla Lana School of Public Health, University of Toronto, Toronto, ON Canada

**Keywords:** Children, Bicycle injury, Risk factors, Health policy

## Abstract

**Background:**

Bicycle injuries are a leading cause of accidental death among children in the world, and bicycle-related injuries are also very common in China, thus to find out bicycle injury risk factors is imperative. This study aims to identify the cyclist-, bicycle- and road-related risk factors of bicycle injury, to develop health education programs as an intervention and to provide a scientific basis for establishing policies against bicycle injury.

**Methods:**

We selected two middle schools randomly among seven schools in Chaoshan rural areas,where the main means of transportation for students from home to school was bicycle. The subjects were middle school students from 7th to 9th grades from Gucuo Middle School and Hefeng Middle School. Cyclists were surveyed through questionnaires about bicycle injury in the past 12 months.

**Results:**

Multivariable logistic analysis showed that compared with a combination-type road、 motor lane and a non-intact road were both risk factors of bicycle injuries. This was followed by riding with fatigue, non-motor lane and inattentive riding.

**Conclusion:**

Bicycle injuries are frequent in China. Three risk factors on bicycle traffic injury among middle school students in Chaoshan rural areas of China were identified. This study provides important data to develop intervention strategies for China and other developing countries.

## Background

Bicycle riding is a popular means of transportation, recreation and exercise for children worldwide [1, 2]. The use of bicycle is often the only way for young students to travel great distances at a fast speed [3]. However, compared to other road users, cyclists run a higher risk of being injured in a road crash [4]. As children have lower awareness of traffic rules and tend to undertake more risky behaviors than adults, bicycle riding is more hazardous for them. Children are much more likely to fall from a bicycle and suffer serious injuries [4]. In the United States, children with 19 years of age and under account for 57% of all bicycle injuries treated in emergency rooms and 15% of deaths. In the US, bicycle accidents are a leading cause of accidental death among children [5]. In developing countries such as China, bicycle is one of the most important means of transportation among teenagers, and consequently, bicycle-related injuries are very common. Li et al. have shown that bicycle-related accidents constitute 71.6% of all types of traffic injuries and fatalities among middle school students [6]. Therefore, effective measures should be taken for prevention of bicycle injury, mainly through interventions on risk factors.

In the review of previous epidemiological studies of cycling-related road crashes, we found that previous studies were usually designed just to investigate the most frequent patterns of accidents, accident circumstances and environment-related factors, or describing the characteristics of injured cyclists and the factors related to injury severity [6, 7]. However, cyclists, vehicles, and roads all play important roles in a bicycle injury. Yet few studies have assessed the cyclist-, vehicle- and road-related factors associated with risk of causing crashes [7]. Moreover, research on bicycle injury were mostly carried out in urban areas and developed countries, and seldom in rural areas of developing countries. To fill this gap, we researched all three factors related to bicycle injuries (i.e. cyclists, bicycles and roads) and investigated major risk factors of bicycle injury in the Chaoshan rural areas of China. We want to provide data which are representative of many underdeveloped areas in developing countries where bicycles are very commonly used among middle school students. The study is expected to provide data for development of intervention strategies for effective prevention and control of bicycle injuries.

## Methods

### Subjects

This study involved a large group of junior high school students, through stratified cluster sampling. The subjects were Middlestudents from 7th to 9th grades from Gucuo Middle School and Hefeng Middle School. These two schools were selected randomly among seven schools in Chaoshan rural areas, where the main means of transportation for students from home to school was bicycle. Only students who stated ‘go to school by bicycle’ were included in this study. We hand out 2421 questionnaires and got 2075 back, they were all valid questionnaires. the effective rate was 85.7% (2075/2421). The prevalence of bicycle injury was 28.3% (588/2075). The injured students ranged from 11 to 19 years old, with an average age of 14.77 ± 1.07.

### Data procedure

Informed written consent was given by the students, or the next of kin, caregivers or guardians on behalf of the minor/children participants involved in this study. Before the investigation, a questionnaire was designed based on the Haddon Matrix theory [8, 9] and modified after a pilot study. Between November 2012 and March 2013, through stratified cluster sampling, students in two middle schools of Liangying Village in Chaoshan rural areas were recruited. Cyclists were surveyed through questionnaires about bicycle injury in the past 12 months. We collected data, including gender, age, basic knowledge of and attitude towards bicycle injury prevention (opinion on necessity of setting up traffic rules, recognition of traffic markings “for vehicles only”, running a red light, opinion on whether bicycle injury is preventable, willingness to learn about prevention of bicycle injuries), lifestyle and bicycle riding behaviors (coffee or strong tea consumption, inattentive riding, riding speed, use of communication devices while riding, riding with fatigue, riding in bad mood), bicycle-related factors (periodic maintenance of bicycle, bicycle age), road-related factors (types of lanes, road situation, road surface, road materials). Validity and reliability were analyzed and the test-retest reliability and validity were both above 0.70.

During the investigation, trained interviewers explained the research purposes, the meaning of questionnaire items, and the filling methods to respondents. The questionnaires were collected and checked. Personal information of the respondents was completely confidential. The information collected was only used for summary analysis.

### Statistical analysis

Respondents were classified into two groups: injured and uninjured. Injured refers to conditions meeting at least one of the following three criteria: 1.going to a medical institution for treatment, and being diagnosed as having an injury; 2. asking for one or more days off school because of an injury; 3. being given first-aid by family members, teachers or others because of an injury.

Using Epi Data 3.1 software, we entered data into a computer database, using double key entry to improve accuracy. Descriptive analysis was first used to study the variables. A chi-squared test was used to study significant differences between the two groups (injured and uninjured). Multivariable logistic analysis was used to assess the impact of variables on bicycle injury. A *P* value less than 0.05 was regarded as statistically significant. All statistical analyses were performed with SPSS 19.0.

### Quality control

We trained interviewers to conduct the questionnaire survey, and applied methods of data collection and analysis in a consistent way. The quality control process of the study was carried out by assigned field coordinators during the course of investigation. The collected survey data was entered into the database after verification and a logical check was executed. The answers of 3% of respondents were verified through a phone call to ensure that the collected data was reliable.

## Results

General conditions of the subjects between the respondents from the two junior high schools in Liangying Village, 85.7% of students went to school by bicycle. General conditions of the surveyed students from the two junior high schools in Liangying Village are given in Table 1. The prevalence of bicycle injury was 28.3%. Among the injured students, there were 357 males (60.7%) and 231 (39.3%) females. Chi-squared analysis comparing the distribution of gender and age in Table 1 suggested that male gender was related to the occurrence of bicycle injury (*P* <0.01, *χ*2 = 47.992). The relation between age and the occurrence of bicycle injury was non-significant (*P* = 0.961).

**Table 1:** General conditions of the middle school students in Chaoshan rural areas of China

Subject		The injured n (%)	Sample size n	Prevalence of injury (%)
Gender^†^				
	Male	357 (60.7)	1009	35.4
	Female	231 (39.3)	1066	21.7
Total		588 (100)	2075	28.3
Age^a^				
	11-13	63 (10.7)	227	27.8
	14-16	497 (84.5)	1745	28.5
	17-19	28 (4.8)	103	27.2
Total		588 (100)	2075	28.3

^†^(*P*<0.01,χ2=47.992), ^a^(*P*=0.961,χ2=0.079)

### Cyclist-related factors

#### Basic knowledge of and attitude towards bicycle injury prevention

Basic knowledge of and attitude towards bicycle injury prevention are presented in Table 2. Almost all the students (88.9%) thought it was highly necessary to set up traffic rules. Students who thought that setting up traffic rules was of little or no necessity had a higher prevalence of bicycle injuries (45.2%) than those who thought that setting up traffic rules was necessary (27.6%). 65.3% of students were willing to learn more about prevention of bicycle injuries. Although more than half (62.2%) of students believed that bicycle injury was preventable, some of the students (28.5%) were unsure whether or not bicycle injury was preventable.

**Table 2:** Basic knowledge of and attitude towards injury prevention among middle school students in Chaoshan rural areas of China

	Whether bicycle injuries occurred			
	Yes (n)	No (n)	Prevalence of injury (%)	*P*
Opinion on necessity of setting up traffic rules				0.004
Highly necessary	504	1340	27.3	
Moderately necessary	46	101	31.3	
Little necessary	18	22	45.0	
Not at all	20	24	45.5	
Recognition of traffic markings “for vehicles only”				0.509
No	425	1096	27.9	
Yes	163	391	29.4	
Running a red light^*^				<0.01
No	545	1439	27.5	
Yes	43	48	47.3	
Opinion on whether bicycle injury is preventable				0.009
Yes	341	925	26.9	
No	63	101	38.4	
Uncertain	184	461	28.5	
Willingness to learn about prevention of bicycle injuries				0.020
No	93	169	35.5	
Yes	366	990	27.0	
No idea	129	328	28.2	

^*^If you come across an intersection with the red light on, but there is no other vehicle around, will you ignore the traffic rules and run red light just to save time?

73.3% of the students could not recognize the traffic markings “for vehicles only”. According to the chi-squared test, the relation between recognition of traffic markings “for vehicles only” and the occurrence of bicycle injury was non-significant (*P* = 0.509). Regarding the question of running a red light (If you come across an intersection with the red light on, but there is no other vehicle around, will you ignore the traffic rules and run the red light just to save time?), there was a higher prevalence among students who chose ‘Yes’ (47.3%) than those who chose ‘No’ (27.5%).

#### Lifestyle and bicycle riding behaviors

Data in Table 3 presents the behaviors and habits of bicycle riding elicited from the subjects. Most of the students (88.8%) rode at a speed between 10 km/h to 20 km/h. For students who rode either faster (≥20 km/h: 79.4%) or slower (≤10 km/h: 55.4%), the prevalence were much higher than those who rode at a moderate speed (>10 to <20 km/h: 23.6%). Students who rode a bicycle when they were sleepy or tired had a higher prevalence rate (40.3%) than those who did not (24.0%). Moreover, negative mood (anger, grief, etc.) was related to bicycle injury. Students who rode while having bad mood were more likely to have bicycle injuries (37.1%) than those who did not (21.2%). Of the 1178 students who usually drank coffee or strong tea in their daily life, 31.7% had experienced bicycle injuries. In contrast, among 897 students who did not drink coffee or strong tea, only 24.0% had been injured in bicycle accidents.

**Table 3:** Lifestyle and bicycle riding behaviors of middle school students in Chaoshan rural areas of China

Have you ever had these behaviors and habits in the past 12 months	Whether bicycle injuries occurred			
	Yes (n)	No (n)	Prevalence of injury (%)	*P*
Coffee or strong tea consumption				<0.01
Yes	373	805	31.7	
NO	215	682	24.0	
Inattentive riding				<0.01
Yes	446	976	31.4	
No	142	511	21.7	
Riding speed (km/h)				<0.01
≥20	81	21	79.4	
>10 to <20	435	1408	23.6	
≤10	72	58	55.4	
Use of communication devices (like mobile phones) while riding				<0.01
Yes	101	141	41.7	
No	487	1346	26.6	
Riding with fatigue				<0.01
Yes	221	328	40.3	
No	367	1159	24.0	
Riding in bad mood				<0.01
Yes	344	582	37.1	
NO	244	905	21.2	

#### Bicycle-related factors

Bicycle-related factors in bicycle injury among 2075 students in Chaoshan rural areas of China is showed in Table 4,which indicate that neither periodic maintenance of bicycle nor bicycle age was significantly related to bicycle injuries.

**Table 4:** Bicycle-related factors in bicycle injury among middle school students in Chaoshao rural areas of China

	Whether bicycle injuries occurred			
	Yes (n)	No (n)	Prevalence of injury (%)	*P*
Periodic maintenance of bicycle				0.593
Yes	51	118	30.2	
No	537	1368	28.2	
Bicycle age				0.398
<3 months	73	151	32.6	
3 ~ 6 months	72	167	30.1	
6 ~ 12 months	91	242	27.3	
>12 months	352	927	27.5	

#### Road-related factors

We asked questions about the road conditions from the students’ home to school, including types of lanes, road situation, road surface and road materials. It shows that 74.9% of students went to school on a combination-type road (roads that are not divided into specific lanes) in Table 5. The prevalence of injury on motor lanes and non-motor lanes were higher than that on combination-type road (motor lane: 43.5%, non-motor lane: 47.8%, combination-type road: 22.1%). In addition, the injury prevalence of students who rode on non-intact roads was higher than those who rode on intact roads (43.1 and 23.3%, respectively). There was also a significant difference among injury prevalence on different road surfaces (dry: 18.8%, ponding: 46.5%, humid: 47.9%). Students riding on roads composed of sand and rocks or asphalt had higher rates of injury (sand and rocks: 50.6%, asphalt: 47.4%) than cement (21.3%).

**Table 5:** Road-related factors in bicycle injury among middle school students in Chaoshan rural areas of China

	Whether bicycle injuries occurred			
	Yes (n)	No (n)	Prevalence of injury (%)	*P*
Types of lanes				<0.01
Motor lane^β^	50	65	43.5	
Non-motor lane^θ^	194	212	47.8	
Combination-type road^**^	334	1210	22.1	
Road situation				<0.01
Intact	362	1189	23.3	
Non-intact^††^	226	298	43.1	
Road surface				<0.01
Dry	261	1131	18.8	
Ponding	182	209	46.5	
Humid	135	147	47.9	
Road materials				<0.01
Cement	335	1235	21.3	
Sand and rocks	217	212	50.6	
Asphalt	36	40	47.4	

^β^lane that only allows motor vehicles, ^θ^lane that only allows pedestrians and bicycles, ^**^road that is not divided into specific lanes, ^††^road that is uneven or under construction

#### Logistic regression analysis on bicycle traffic injury among middle school students

We performed univariate logistic regression analysis and set the occurrence of bicycle injury as the dependent variable, and cyclist-, bicycle- and road-related factors as the covariates. Chi-squared results showed that male gender (*P* <0.01), opinion on necessity of setting up traffic rules (*P* = 0.004), running a red light (*P* <0.01), opinion on whether bicycle injury is preventable (*P* = 0.009), willingness to learn about prevention of bicycle injuries (*P* = 0.020), coffee or strong tea consumption (*P* <0.01), inattentive riding (*P* <0.01), riding speed (*P* <0.01), use of communication devices (like mobile phones) while riding (*P* <0.01), riding with fatigue (*P* <0.01), riding in bad mood (*P* <0.01), types of lanes (*P* <0.01), road situation (*P* <0.01), road surface (*P* <0.01), and road materials (*P* <0.01) were related to bicycle injury (Table 1, Table 2, Table 3, Table 4, Table 5).

5 statistically significant risk factors with *P* <0.01 were put into Multivariable logistic regression analysis. Odds ratios (OR) calculated for variables showed that the most important risk factors were: riding on motor lanes (OR = 2.414, 95% CI = 1.474 ~ 3.954), non-intact road situations (OR = 2.261, 95% CI = 1.632 ~ 3.133), riding with fatigue (OR = 1.714, 95% CI = 1.196 ~ 2.458), riding on non-motor lanes (OR = 1.648, 95% CI = 1.161 ~ 2.339) and inattentive riding (OR = 1.599, 95% CI = 1.122 ~ 2.278). (Table 6).

**Table 6:** Multivariable logistic regression analysis on bicycle traffic injury among middle school students in Chaoshan rural areas of China

Variable factor	*P*	OR	95%CI
Inattentive riding			
No	-	1.0	Reference
Yes	0.009	1.599	1.122 ~ 2.278
Use of communication devices (like mobile phones) while riding			
No	-	1.0	Reference
Yes	0.051	0.605	0.366 ~ 1.002
Riding with fatigue			
Yes	-	1.0	Reference
No	0.003	1.714	1.196 ~ 2.458
Road situation			
Non-intact	<0.01	2.261	1.632 ~ 2.339
Intact	-	1.0	Reference
Types of lanes			
Non-motor lane	0.005	1.648	1.161 ~ 2.339
Motor lane	<0.01	2.414	1.474 ~ 3.954
Combination-type road	-	1.0	Reference

See footnotes to Table 5

## Discussion

### Cyclist-related factors

A previous study [10] showed that the prevalence of bicycle injury among junior high school students in the Yangpu District of Shanghai, China is 8.71%. In the current research, the prevalence of bicycle injury in Chaoshan rural areas is 28.3%, which is much higher than that in the city. Therefore, bicycle injury prevention is a critical issue in the rural areas of China. Many studies show that younger age is a risk factor of bicycle injury. For instance, in America, one-fifth of bicycle-related injuries occurs in children 15 years and younger [11] with peak prevalence of bicycle-related injuries and deaths occurring within the 9–15 years-old age group [12]. Another study also found that risk of serious injury is increased by young age (<6 years) [13]. However, in this study, the prevalence of bicycle injury in different age groups (11–13, 14–16, 17–19) are nearly the same (27.8%, 28.5%, 27.2%, *P* = 0.961, Table 1). Therefore, a bicycle education program should focus on junior high students of all age groups, and not only the younger students.

Being male is associated with an increased risk for all types of injuries [14]. This study showed that the prevalence of injury in males (60.7%) is higher than that in females (39.3%).This supports previous research on bicycle injuries of children, where a male-to-female ratio of 3:1 has been reported [2]. This may be related to the nature of males. Firstly, males tend to have more impulsive, un-controlled behavioral styles that lead to a higher risk of unintentional injury [15]. Secondly, males are more likely to attribute injuries to bad luck, and females to their own behaviors and decisions, leading males to repeat injury risk behaviors more often than females [16]. So we should pay more attention to male students in order to reduce their high injury rate. Parental supervision is among the most effective behavioral techniques for reducing pediatric injury risk [17]. Adult supervisors can verbally intervene when male students begin to behave in a dangerous way. Supervision causes un-controlled children to reduce their tendency toward overestimation of ability and to judge their abilities more cautiously [18]. Moreover, bicycle injury education should be emphasized, especially for male students, in order to correct their wrong perspective (e.g. attributing injuries to bad luck) about injury. Also, the Bike Smart program (an eHealth product that utilizes video, animations, and still images to train children to acquire the key skills for bicycle safety) is a good way to improve males’ cycling skills, because studies suggest that Bike Smart can be a low cost, effective component of safety training packages that include both skills-based and experiential training [12]. A walking school bus (a group of children walking to school with one or more adults) is a proven way to reduce cycling time, but few statistics are available to show whether it is really safer for children in China [19].

### Basic knowledge of and attitude towards bicycle injury prevention

Attitudes towards bicycle safety play an important role in predicting child safety practices [20]. In this study, we investigated the attitudes of students on bicycle injuries (opinion on necessity of setting up traffic rules, opinion on whether bicycle injury is preventable, and willingness to learn about prevention of bicycle injuries). Results show that students who had a positive attitude towards bicycle injury prevention (thinking that setting up traffic rules was necessary, feeling that bicycle injury is preventable, and willing to learn about prevention of bicycle injuries) had a lower prevalence of injury than those who did not. Also, a previous study has suggested that better road safety knowledge is a protective factor for road traffic injuries among Chinese school children [21]. Specifically, a bicycling education program implemented in selected New Jersey schools and summer camps has shown that using road sign identification as an educational item in the program is effective in reducing bicycle crashes [22]. In our study, traffic marking recognition “for vehicle only” was also included. However, we found that it was only of borderline significance on bicycle injury (*P* = 0.509) according to chi-squared test. Why the result of our study was different from that of the New Jersey study could relate to the fact that there are few traffic signs on roads in Chaoshan rural areas, so even if students could recognize the markings in the questionnaire, it might not prevent them from bicycle injuries because they seldom encounter traffic markings on the road. Therefore, bicycle education programs focusing on recognition of traffic markings “for vehicles only” are not recommended before traffic signs are well established on the roads.

### Lifestyle and bicycle riding behaviors

Accidents involving child cyclists are often the result of the children playing, doing tricks, riding too fast or losing control [23]. These behaviors may sometimes help children gain experience and training in bicycle handling skills. However, practicing good habits is more important to enhance safety. Also, caution and rational behaviors should be emphasized more on the roads. A study in Taipei shows that speeding is a factor related to severe head injury (*P* = 0.031) [24]. In our study, while riding at a high speed (≥20 km/h) is correlated with a higher prevalence of injury (79.4%), riding at a low speed (≤10 km/h) is also related to a higher rate of injury (55.4%), compared to those riding at a moderate speed (>10 to <20 km/h, 23.6%). Slower speed may be associated with longer exposure time. Thus we advise students to ride at a moderate speed (>10 to <20 km/h), rather than low (≤10 km/h) or high (≥20 km/h) speeds, for prevent bicycle injury prevention.

The data shows that coffee or strong tea consumption is related to bicycle injuries. it can make the riding students more excited, and cause the bicycle injury. Therefore, avoiding coffee and strong tea consumption is recommended for bicycle injury prevention. Injured students show bad habits, such as using communication devices (like mobile phones) while riding. This result supports prior research from Japan, which indicates that there is a significant relationship between phone usage while riding a bicycle and bicycle crash/near-crashes [25]. Also, mental status could play a role in bicycle injury. Being sleepy or being in bad mood (anger, grief, etc.) while riding could easily distract the riders and thus increases the risk of accidents. In order to reduce both the occurrence and severity of bicycle injury, all of these bad habits need to be avoided. In addition, law enforcers should do their job to prevent harmful behaviors in the first place, such as using mobile phones [26].

In this study, 65.3% of the students were willing to acquire more knowledge on bicycle injury prevention. Teachers, parents or other close relatives of children are recommended to be educated on bicycle injury prevention so as to pass on the knowledge to children to prevent potential injuries. More importantly, teachers and parents should set a good example by their own actions, such as traffic rule obedience, being cautious and attentive in using bicycles, etc.

### Bicycle-related factors

Our research reveals that 8.1% of 2075 students perform regular bicycle maintenance (Table 4). Prior research shows that several defects result in single-bicycle crashes (i.e. a fall or obstacle collision): the chain broke off, the tire inflation was too low resulting in skidding while cornering, the fender or the front fork broke off, a wheel, saddle, or handlebars were loose or broke off [27]. However, in this study, there was no significant difference between the prevalence of bicycle injuries among students who maintained their bicycles periodically and those who did not (*P* = 0.593). Actually, the former group has slightly higher injury prevalence (30.2%) than the latter group (28.2%). The reason is probably that students who experience bicycle injuries care more about their bicycles, and thus are more likely to maintain their bicycles periodically after bicycle injury. Further research is required to identify whether there is a difference in habits of bicycle maintenance before and after injury and, also, whether bicycle injury prevalence changes before and after the formation of this habit. We do not recommend junior high school students to replace their old bicycle with a new one in order to prevent bicycle injury because we find that bicycle age is not related to bicycle injury prevalence (*P* = 0.398), and the prevalence of injury for different bicycle age groups are similar, around 30%.

### Road-related factors

According to research done in Hungary, terrible road conditions are among the most important causes of bicycle traffic injury among rural children [2]. Our research finds that riding on a motor lane (OR = 2.414, 95% CI = 1.474 ~ 3.954) is a risk factor for bicycle injury. Riding on a motor lane violates traffic rules and increases the risk of a bicycle crash by motor vehicles. Previous research also indicates that in bicycle accidents, involvement in a collision with a motor vehicle increases the risk of severe injury by 3.6-fold [28].

The crash involvement rate per 1000 cyclists has been shown to be 11.8 on shared paths compared to 5.8 on cycle lanes [29]. It has been suggested that having a bicycle-friendly infrastructure (e.g. bike routes, painted bike lanes) is associated with a low injury risk [30]. Road infrastructure that separates motor vehicles from bicycles is common in some cycling countries [31, 32]. Providing a one-way bicycle path for bicycle users can reduce the chance of severe injury [33]. However, in this study, riding on a non-motor lane (OR = 1.648, 95% CI = 1.161 ~ 2.339) is also a risk factor for bicycle injury compared with riding on a combination-type road. We attribute this finding to the fact that in rural areas of China, roads are seldom divided into specific lanes. All types of vehicles ride on the same lane (combination-type road). Therefore, cyclists are more cautious to avoid crashes with vehicles when they are on this type of road. On the contrary, cyclists may not be highly alert on non-motor lanes, thinking that these lanes are much safer. However, large numbers of drivers may not obey traffic rules and may take up the whole non-motor lane for convenience. Accidents happen under these circumstances. Therefore, separating motor vehicles from non-motor ones may help to improve cyclist safety in urban areas. But in Chaoshan rural areas of China specifically, more education is required to improve road users’ awareness of traffic rule obedience and accident prevention before non-motor lanes can really play a role in bicycle injury prevention. Moreover, the traffic department should set up an effective reward and punishment system that would contribute to better administration of traffic rules and regulations.

In addition, insufficient infrastructure of rural roads and terrible road conditions are related to higher rates of bicycle injury in rural China. We show that non-intact road situations (OR = 2.261, 95%CI = 1.632 ~ 3.133) are important risk factors for bicycle injuries. The prevalence of students who ride on non-intact roads is much higher than those who ride on intact roads (43.1% and 30.4%). Moreover, compared to dry road surfaces, ponding or humid road surfaces are related to a higher prevalence of bicycle injuries (dry: 18.8%, ponding: 46.5%, humid: 47.9%). There is also a significant difference among prevalence on different road materials. Roads composed of sand and rocks, as well as asphalt, are associated with a higher bicycle injury rate compared with cement roads (sand and rocks: 50.6%, asphalt: 47.4% cement: 21.3%). the students are used with riding on the cement roads, which are most common in rural China. In summary, intactness, dryness and cement road play essential roles in bicycle injury prevention. All of these factors should be managed by the government. Government intervention can be effective in lowering the rates of injury [34]. In Australia, bicycle-related head injuries have steadily declined in the years after 2006, the year associated with an increase in spending on cycling infrastructure [35]. According to this research, the government must take measures to improve road conditions, which include repairing broken and uneven roads, separating road sections that are under construction with warning signs, equipping roads with effective drainage systems, and paving roads with cement instead of sand and rocks. Much work still needs to be done.

### Study limitations

In this study, the accuracy of self-reports may be compromised because some health-risk behaviors are difficult to recall and some are so sensitive that respondents may not want to report them. Also, the use of self-report data on road traffic risk behaviors and injuries may be subject to social desirability biases [36]. Moreover, in recalling what happened, the injured cyclists usually examine their own behaviors more critically. Thus, they are more likely to admit to negative behaviors than the uninjured cyclists. Considering the reality of Chaoshan rural areas of China, where students hardly wear a helmet to go to school, we excluded the questions on helmet usage, although in foreign countries, it has been proven that a primary strategy to prevent bicycle-related injuries is the promotion of bicycle helmet use [37]. In addition, exposure time of bicycle injuries and the cluster effect are two factors which should be taken into consideration [38]. This current research does not cover these parts and we will take them for the further research.

## Conclusion

Multivariable logistic analysis showed that compared with a combination-type road、 motor lane and a non-intact road were both risk factors of bicycle injuries. This was followed by riding with fatigue, non-motor lane and inattentive riding. the study investigate the epidemiological characteristics of bicycle injuries in the region and the related risk factors for the issue.

## Abbreviation

Combination-type road: Road that is not divided into specific lanes
Motor lane: Lane that only allows motor vehicles
Non-intact road: Road that is uneven or under construction
Non-motor lane: Lane that only allows pedestrians and bicycles
